# Poster Session I - A134 EVALUATING ENDOSCOPIC RETROGRADE CHOLANGIOPANCREATOGRAPHY (ERCP) PANCREATITIS PREVENTION MEASURES AMONGST TWO TERTIARY CARE CENTERS IN WINNIPEG, MANITOBA: A QUALITY IMPROVEMENT STUDY

**DOI:** 10.1093/jcag/gwaf042.134

**Published:** 2026-02-13

**Authors:** G S Sambhi, J G Coneys, D Moffatt, J K Stone

**Affiliations:** Gastroenterology, University of Manitoba Max Rady College of Medicine, Winnipeg, MB, Canada; Gastroenterology, University of Manitoba Max Rady College of Medicine, Winnipeg, MB, Canada; Gastroenterology, University of Manitoba Max Rady College of Medicine, Winnipeg, MB, Canada; Gastroenterology, University of Manitoba Max Rady College of Medicine, Winnipeg, MB, Canada

## Abstract

**Background:**

Two tertiary care centers provide ERCP services for the entire province of Manitoba. These centers differ in pre-ERCP indomethacin protocols. At Health Sciences Center (HSC) it is endoscopist driven and must be requested while at St. Boniface Hospital (SBH) it is part of a pre-ERCP nursing checklist. Assessing ERCP metrics ensures adherence to evidence-based best practices and minimizes procedural complications.

**Aims:**

Conduct a quality improvement study comparing two tertiary care centers in Manitoba to evaluate for differences in pancreatitis prevention measures.

**Methods:**

A retrospective chart review was conducted on all ERCPs performed by the three therapeutic endoscopists from January 1, 2025 to September 30, 2025. Data collected included patient age and sex, presence of a native papilla (NP), common bile duct (CBD) cannulation rate and difficulty (as defined by the 2016 ESGE ERCP guidelines), biliary stent placement rate, pancreatic duct (PD) cannulation rate and PD stent placement rate.

**Results:**

Between January 1, 2025 and September 30, 2025, 753 ERCPs were performed (n = 645 at SBH, n = 108 at HSC). The rectal indomethacin administration rate was higher at SBH (97.4% vs. 93.5%, p = 0.07). There was a significantly higher rate of PD stent use in patients with any PD cannulation at SBH (81.3% vs. 57.9%, p = 0.02). When assessing only those with ≥2 PD wire passes in patients with a NP, overall rates were >90%, favoring SBH (94.5% vs. 91.6%, p = 0.69). NP cannulation rates were similar at both sites (95.8% vs. 94.9%, p = 0.72). The most common indications for ERCP at SBH were CBD stones (45.3%) and stent removal (9.1%). At HSC they were CBD stones (38.0%) and bile duct obstruction (15.7%).

**Conclusions:**

ERCP pancreatitis prevention quality metrics were similar between the two centers. A higher proportion of patients received rectal indomethacin at SBH, although this was not statistically significant. We have applied for ethics approval to investigate post-ERCP pancreatitis rates in this cohort. Based on these findings, we plan to propose the implementation of a standardized pre-ERCP checklist at HSC.

A127 Table 1: ERCP metrics at SBH and HSC

**Funding Agencies:**

None

**Table:** 

	SBH (n = 645)	HSC (n = 108)	P-Value
Rectal Indomethacin Rate,%	97.4	93.5	0.07
NP Cannulation Rate,%	95.8	94.9	0.72
Difficult CBD Cannulation,%	19.8	14.8	0.22
Rectal Indomethacin Rate for Difficult CBD Cannulation,%	98.4	100.0	1.00
NP PD Cannulation Rate,%	25.6	21.5	0.44
PD Stent Rate in NP Patients with PD Cannulation,%	82.7	64.7	0.08
PD Stent Rate in NP Patients with PD Cannulation Adjusted For ≥2 PD Wire Cannulations,%	94.5	91.6	0.69
PD Stent Rate in Patients with any PD Cannulation,%	81.9	57.9	0.02
PD Cannulation Rate for Difficult CBD Cannulation,%	51.2	62.5	0.39
PD Stent Rate in Patients with PD Cannulation and Difficult CBD Cannulation,%	90.9	90.0	0.94

